# Prostate cancer and toxicity from critical use exemptions of methyl bromide: Environmental protection helps protect against human health risks

**DOI:** 10.1186/1476-069X-11-5

**Published:** 2012-01-27

**Authors:** Lygia T Budnik, Stefan Kloth, Marcial Velasco-Garrido, Xaver Baur

**Affiliations:** 1 Division of Occupational Toxicology and Immunology, Institute for Occupational and Maritime Medicine (ZfAM), Medical Faculty, University of Hamburg, University Medical Center, Hamburg, Germany; 2 Division of Clinical Occupational Medicine, Institute for Occupational and Maritime Medicine (ZfAM), University Medical Center Hamburg-Eppendorf, Hamburg, Germany; 3 Chair for Occupational Medicine, Medical Faculty, University of Hamburg, University Medical Center, Hamburg, Germany

**Keywords:** methyl bromide, bromomethane, fumigant, halomethane, pesticide, toxic effect, carcinogenic risk, critical use exemptions

## Abstract

**Background:**

Although ozone-depleting methyl bromide was destined for phase-out by 2005, it is still widely applied as a consequence of various critical-use-exemptions and mandatory international regulations aiming to restrict the spread of pests and alien species (e.g. in globalized transport and storage). The withdrawal of methyl bromide because of its environmental risk could fortuitously help in the containment of its human toxicity.

**Methods:**

We performed a systematic review of the literature, including in vitro toxicological and epidemiological studies of occupational and community exposure to the halogenated hydrocarbon pesticide methyl bromide. We focused on toxic (especially chronic) or carcinogenic effects from the use of methyl bromide, on biomonitoring data and reference values. Eligible epidemiological studies were subjected to meta-analysis.

**Results:**

Out of the 542 peer reviewed publications between 1990-2011, we found only 91 referring to toxicity of methyl bromide and 29 using the term "carcinogenic", "neoplastic" or "mutagenic". Several studies provide new additional data pertaining to the mechanistic aspects of methyl bromide toxicity. Few studies have performed a detailed exposure assessment including biomonitoring. Three evaluated epidemiological studies assessed a possible association between cancer and methyl bromide. Overall, exposure to methyl bromide is associated with an increased risk of prostate cancer OR, 1.21; 95% CI (0,98-1.49), P = 0.076. Two epidemiological studies have analyzed environmental, non-occupational exposure to methyl bromide providing evidence for its health risk to the general public. None of the epidemiological studies addressed its use as a fumigant in freight containers, although recent field and case reports do refer to its toxic effects associated with its use in shipping and storage.

****Conclusion**s:**

Both the epidemiological evidence and toxicological data suggest a possible link between methyl bromide exposure and serious health problems, including prostate cancer risk from occupational and community exposure. The environmental risks of methyl bromide are not in doubt, but also its health risks, especially for genetically predisposed subjects, should not be underestimated.

## Background

Fumigation with pesticides is a widely used defensive measure against the multitude of pests responsible for destroying foodstuffs and other natural commodities during storage and transport. Necessarily, pesticide chemicals are highly toxic to pests, but present also a substantial risk to both human health and the environment [1-5]. The methyl and ethyl halides, in particular methyl bromide (IUPAC name: bromomethane), are highly effective fumigants and are often used as pesticides, both during and after the harvest. Methyl bromide is a broad spectrum pesticide with a long history of use as a fumigant in farming (stripping the soil of pathogens) and for disinfecting furniture, wood, barges, warehouses, buildings and cargo ships [1-3,5]. Its use has accelerated more recently because of increased globalization and the perceived threat of invasion by alien species. Recent regulations requiring fumigation with methyl bromide (or heat treatment) of wooden packaging, flooring and wooden goods in imported freight containers [6] have resulted in an epidemic of freight container fumigation.

To be set against the desirable characteristics of this almost perfect fumigant is its remarkable potency as a depleter of atmospheric ozone. Methyl bromide and related ozone-depleting compounds were banned in the 1987 Montreal and 1997 Kyoto Protocols [4] and methyl bromide was destined for a phase-out of production within the current decade (2005 by industrial nations and 2015 by developing nations). The ocean is a net sink for atmospheric methyl bromide, where it is slowly degraded by chemical and biological processes [7].

Although more than 15 industrialized nations have claimed not to fumigate with methyl bromide anymore, most continue to do so under the auspices of a critical use exemptions (CUE) clause. The CUE allows continued use of methyl bromide where no adequate alternative is available, thus assuring its unremitting popularity and widespread use as a fumigant. In 2003, methyl bromide was the most commonly used pesticide among California growers [8,9] and since 2001 it is required for fumigation of grapes in the US [10]. This pesticide is still being used in agriculture [11], in urban pest control [12], and for processing onboard ship [13-16]. Also in major ports worldwide, several hundred tons of methyl bromide continue to be used annually for the fumigation of containers destined for export, representing a substantial environmental and human health risk [17-19]. Fumigation of freight containers with methyl bromide is a standard procedure, particularly in Asia [17,18], though adequate alternatives like heat treatment are known. The imported containers and the fumigated products are shipped deep within an importing country before being opened, unloaded, distributed and used by workers and the general public. The primary routes for methyl bromide exposure are by inhalation and by dermal absorption from direct skin contact [20]. Exposure due to off gassing is likely since methyl bromide persists on clothes, leather, and rubber brought home or when entering storage facilities where highly fumigated products are stored [3,11,21]. The most common consequences of a transient exposure to methyl bromide are nervous system symptoms, including headache, nausea, vomiting, dizziness, blurred vision, impairment of coordination and twitching. Acute massive or prolonged exposure ultimately leads to permanent debilitation or death [22]. A link between methyl bromide exposure and cancer has been demonstrated experimentally and is also documented clinically, which is not surprising considering its recognized genotoxic effects [23-25]. From animal studies, the National Institute for Occupational Safety and Health (NIOSH) lists methyl bromide as a potential occupational carcinogen [1,4]. However, the interpretation of toxicological data is often limited by various shortcomings in the available studies. First, the hazard data from animal experiments may not always be immediately relevant to human beings because of the acknowledged physiological and catabolic differences in methyl bromide activity [24]. In addition, several epidemiological studies are vague about the actual pesticide(s) under investigation. Furthermore, inadequate exposure assessment precludes the efficient identification of any causal inferences between a given pesticide and subsequent cancer [25].

For the current study, we performed a systematic review of the literature addressing the risks associated with the exposure to methyl bromide, including the available in-vitro toxicology assessments, in-vivo animal experiments and population-based epidemiological studies. We provide evidence that this pesticide should be phased out not only because of environmental concerns but also because of its human health risks.

### Methods

A Pubmed search for peer-reviewed studies on methyl bromide was performed for the period 1990-2011 [26]. Several combinations of the following MeSH terms were utilized in the search: "methyl bromide", "bromomethane", "halogenated hydrocarbon pesticide", "fumigant", "poisoning", "toxicity", "cancer", "neoplasm" "mutagenic" and "tumour". We selected studies according to the following inclusion criteria:

• original studies published in English or German between June 1990 and July 2011

• in-vivo and in-vitro studies on the toxicity of methyl bromide

• papers analyzing molecular mechanisms underlying possible links between methyl bromide exposure and toxic or cancer risk

• cohort or case-control studies analyzing the association between exposure to halogenated hydrocarbon methyl bromide used as fumigant and the incidence of cancer (any site of cancer)

• studies providing data on exposure assessment and bioavailability.

The results from in vitro and in vivo toxicity studies are summarized in an evidence table and discussed further. The results from the included epidemiological studies were summarized quantitatively. Summary odds ratio (OR), with its corresponding 95% confidence interval, was calculated using both fixed and random effects models [26,27]. We calculated I^2 ^to assess the degree of heterogeneity across studies. Values of I^2 ^under 25% indicate low, up to 60% medium, and over 75% considerable heterogeneity [27]. Meta-analysis results are presented as a forest plot. All calculations were performed with the software Comprehensive Meta-Analysis 2.0. (Biostat™, Englewood, USA).

## Results

The initial electronic database search yielded 543 publications on methyl bromide. 442 were considered not relevant for the review (because they considered the chemical synthesis of methyl bromide, its bacterial or chemical degradation, pest control issues and regulations or did not contribute new information). Among the included studies, 91 matched the terms toxic, toxicological effects or poisoning and 30 matched the terms cancer or DNA damage. We identified only 5 publications reporting epidemiological studies addressing an association between methyl bromide exposure and cancer or toxicity. Two publications reported data from the same study [28,29], three studies addressed the risk of prostate cancer [28,30,31] and were included in the meta-analysis. One additional epidemiological study analyzed the toxic effects of methyl bromide only, but did not report on possible carcinogenic effects [32] and a further one only considered safety issues [33].

### Toxicity of methyl bromide

Methyl bromide, like other methyl halides (i.e. methyl chloride, methyl iodide), has pronounced acute and chronic toxicity (EPA toxicity class I) [4]. It is known as a developmental, neurologic and respiratory toxin [34,35]. Other known target organs are the heart, adrenal glands, liver, kidneys and testis [24]. Chronic low exposure to methyl bromide causes depression of the central nervous system and injury to the kidney. Methyl bromide is a dangerous cumulative poison with the initial symptoms from damage of the nervous system often delayed by 48 hours to several months. The symptoms of acute poisoning vary depending on the concentration and duration of exposure. In sublethal poisoning, the most serious effects involve the central nervous system (with first symptoms including headache, nausea, vomiting, dizziness, malaise and visual disturbances, followed by peripheral neuropathies or neuropsychiatric abnormalities (Table 1). Throat irritation, chest pain and shortness of breath are the most likely first respiratory symptoms with inflammation of the bronchi or lung edema after severe acute exposure. Death may result from respiratory and cardiovascular failure [13,22].

**Table 1:** Toxic effects of methyl bromide (data 1990-2011)

Effect observed	**Ref**.
**in vitro**	

chromosomal aberration (mammalian cells exposed to gaseous methyl bromide)	[54]

Sister chromatid exchange and chromosome aberrations in lymphocytes	

O-6-alkylguanine-DNA-alkyltransferase	[49]

genotoxic in bacteria (Ames test)	[23]

Genotoxicity in workers exposed to methyl bromide	[88]

**in vivo**	

toxic encephalopathies (animal experiments)	[65]

immunoreactive HSP 70 in rat olfactory receptor neurone	[64]

DNA methylation (rat, mice)	[48]

reduction in the white blood cells (rat)	[89]

increase in SCOT, SGPT activities (mice)	[89]

hepatic and glomerular injuries (mice)	[89]

MMP-9, matrix-metalloproteinase -9 and -2, MMP-2 expression in olfactory bulb following methal bromide gas exposure (mice)	[66]

**human**	

irritation of eyes, skin, respiratory system; muscle weakness, coordination loss, visual disturbance, dizziness; nausea, vomiting, headache; malaise (vague feeling of discomfort); hand tremor; convulsions; dyspnea (breathing difficulty); skin vesiculation; liquor frostbite; [potential occupational carcinogen]	[11,34]

acute poisoning: ataxia, behavioral changes, seizures, coma chronic low level exposure: peripheral neuropathy, electroencephalogram abnormalities, deficits on the Wechsler memory scale (on 2-point discrimination at the index scale)	[90]

headache, dizziness, nausea	[11,34]

chronic exposure: central and peripheral system disorders, cerebro-vestibular and pyramidal neuropathy of lower	

limbs, paresthesia cerebro-vestibular and pyramidal neuropathy of lower limbs, paresthesis	

motor neuron disease	[16]

acute exposure (high concentration): refractory seizures, intermittent fever, multiorgan system failure, death	[13]

liver degenerative changes	[1]

reduction of lung function, chest pain, shortness of breath, inflammation of the lung	[1]

erectile dysfunction	[38]

central nervous system toxicity and early peripheral neuropathy following dermal exposure	[36]

diffuse lesions in the spleen of the corpus callosum	[91]

Chronic and acute exposure to methyl bromide may cause respiratory problems, and irritate the skin and eyes. Central nervous system toxicity and early peripheral neuropathy following dermal exposure to methyl bromide [36] confirm the earlier data (see below). Central neurological disorders and chronic toxic encephalopathy were documented in Korean workers after exposure to methyl bromide [37]. Other studies describe motor neuron disease [16], cerebro-vestibular and pyramidal neuropathy, and paresthesia (see Table 1 for details). One clinical case report implicates erectile dysfunction in humans [38].

Structurally similar ethyl halides (i.e. ethylene dichloride, ethyl chloride, ethyl bromide) show less acute toxicities than their methyl counterparts, but more pronounced chronic toxicity [24].

The effects of methyl bromide on regional brain glutathione-S-transferase has been well documented [39]. Human data from accidental poisoning show that the conjugator status plays an important role in the expression of toxicity in humans, with non-conjugators being apparently relieved of the acute neurotoxic effects (see below for more details). They may not be subjectively aware of the toxic exposure, which may lead them into a false sense of security, especially as silent genotoxic effects may only become clinically manifest years after exposure [40-43].

### Genotoxic and carcinogenic effects of methyl bromide

Methyl bromide is genotoxic in vitro, as shown in bacteria [23], animals [44] and human cell culture tests [45] (Table 1). The strong alkylating potency of methyl bromide is primarily responsible for its cytotoxic effect, causing this pesticide to be classified as a potent stimulator of cell growth and, therefore, a potential tumor promoter. Distinguishing alkylation from metabolic incorporation provides proof for the direct genotoxic effect of methyl bromide, methyl iodide and other methyl halides [46-48]. Based on in-vivo and in-vitro studies, methyl bromide induces gene mutations in bacteria, mice and humans. No systemic genotoxic effect was seen with methyl chloride [46,47] in animal experiments. Effects such as DNA single strand breaks after methyl halide intoxication can, however, point to both genotoxic as well as non-genotoxic mechanisms [24]. Methyl bromide causes DNA methylation in rats and mice with concominant decreases in the activity of O^6^-alkylguanine-DNA-alkyltransferase [48]. Interestingly more recent data show that O^6^-alkylguanine-DNA-alkyltransferase has opposing effects in modulating the genotoxicity of dibromomethane, suggesting a pathway which is alternative to the well-recognized pathway that involves activation by GSTs [49]. Conversely, deficiencies in nucleotide excision repair have been shown to strongly potentiate the mutagenic effects of methyl bromide [44]. A clear DNA-alkylating potential of methyl bromide can be demonstrated directly with [^14^C]-methyl bromide binding to DNA in various animal studies [24]. Three additional methylated bases (3-methyl-adenine, 7-methyl-guanine, O^6^-methyl guanine) were also recognized along with further unidentified DNA adducts found in liver, lung and stomach [46]. DNA single strand breaks, liberation of reactive oxygen species and enhanced cell proliferation were detected both in vivo (animal studies) and in vitro using cell-based assays [24,50]. Older studies reported that methyl bromide induces squamous cell papillomas and carcinomas in the forestomach of the rat [4,46]. No carcinogenic effect was observed in further studies applying methyl bromide orally with gavages [51]. A technical report from the US National Toxicology Program showed no evidence of carcinogenic activity in mice exposed to methyl bromide by inhalation [52]. Bolt and Gansewendt [24] explained the negative results in animal experiments by the different or deficient catabolic conjugation pathways for methyl bromide in different species. They also considered that the conclusions from these animal experiments could not be extrapolated to human non-conjugators, since these particular individuals are unable to metabolize methyl bromide as quickly as a rodent can [24]. Other studies report pre-carcinogenic sister chromatid exchange and the induction of chromosome aberrations after exposure to methyl bromide [53,54].

Recent data from Koutros et al. has highlighted the association between the single nucleotide polymorphisms (SNP) in genes coding for xenobiotic-metabolizing enzyme (enzymes of oxidative stress and phase I/II enzymes) and the risk of prostate cancer after exposure to pesticides [55]. The authors could link the enhanced prostate cancer risk after methyl bromide exposure with a SNP in rs93322959 gene coding for the microsomal GST1 enzyme (OR, 3.1; 95% CI (1.3-7.5) and SNP in rs5764318 of cytosolic sulfotransferase, SULT4A1 (OR, 2.2; 95% CI (1.0-4.5). Such polymorphisms may lead to an imbalance in the oxidative stress/antioxidant status, resulting in DNA/chromosome damage and/or induction of possible epigenetic or tumor suppressor gene alterations [55].

### Possible molecular mechanisms

**Figure 1:**
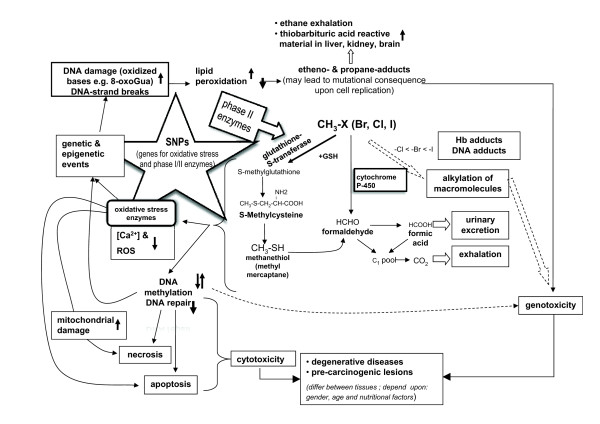
**Scheme summarizing available literature data on possible molecular mechanisms of methyl bromide effects leading to either degenerative diseases or pre-carcinogenic lesions**.

According to the alkylation hypothesis, the methylating activity of methyl bromide should play an important role in the molecular mechanism of toxicity for methyl bromide. Besides this, epigenetic damage [56] may be the most important fundamental cause of degenerative diseases and it can induce carcinogenic lesions (see Figure 1 for a simple model summarizing the current knowledge on non-linear response relationships between the exposure to halomethane methyl bromide, oxidative stress status, DNA damage and pre-carcinogenic lesions).

The conjugation with glutathione, is regarded as the main initiation pathway of methyl bromide: upon inhalation of [^14^C]-methyl bromide, some radioactivity was covalently attached to haemoglobin [57]. The presence of S-methyl-cysteine in the haemoglobin of workers exposed to methyl bromide has been demonstrated [42]. Humans accidentally exposed to either methyl bromide, methyl iodide, methyl chloride in Japan, The Netherlands or in the US showed similar S-methyl-cysteine levels after exposure suggesting similar metabolism of methyl halides in older literature.

Although metabolism of methyl bromide, methyl chloride and methyl iodide has been studied in different systems and to different extents, it has been suggested that the general metabolic scheme is valid for all methyl halides. The tissue specificity and the degree of toxicity of the organic halides are manifested either by the parent compound or their metabolic or catabolic products. The genotoxic effects of methyl bromide appear to be caused by the direct alkylation of macromolecules, producing adducts [40] and sister chromatid exchange [41]. Conversely, the neurotoxic effects appear to arise after the alkylation of methyl bromide by conjugation with glutathione, producing acutely toxic catabolites that preferentially target the nervous system [42].

Data collected within the last five years point to an intriguing association between the alkylation activity of methyl bromide (which is modulated by the expression of various isoforms of GST) and the development of prostate cancer. Two gene products seem to be involved in the epigenetic changes caused by methyl bromide. Pi-class glutathione S-transferases (GSTP1) protect the cell from cytotoxic and carcinogenic agents and have been found to be hypermethylated and silenced in prostate cancer tissue [56,58]. The glutathione *S*-transferase *theta *(GSTT1) gene, whose activity can be influenced by methyl bromide in human erythrocytes, was reported to be positively associated with the risk of prostate cancer [59-61], although other studies have not found these associations [62,63]. It must be pointed out that glutathione S-transferases may also undergo complex epigenetic changes, such as hyper/hypomethylation, depending on the stage of the carcinogenic progression of the prostate cancer.

The molecular mechanisms responsible for the neurotoxic effects of methyl bromide (either alone or with other halo-methanes or halo-ethanes) have been elucidated to great extent [50]. Methyl halides (and probably also ethyl halides) readily react with GST causing its depletion in several cerebellum cell types and lowering the antioxidant status of these cells [50]. There is a marked cooperation between neurones and astrocytes with regard to maintenance of GSH. GSH is toxic to isolated cerebellar granule cells in culture and to astrocytes. The mechanism of neuronal cell loss with methyl halides appears to involve DNA damage, methylation and inhibition of DNA repair, plus depletion of the intracellular antioxidant GSH and oxidative stress; the apoptotic pathways and neuronal cell death may be switched on [50].

Additionally, recent data provide evidence for the mechanistic aspects of methyl bromide neurotoxicity and point to its ability to alter epithelial density and expansion of bulbar projections [64], to inhibit creatine kinase in rat brain [65] or its effects on matrix metalloproteinase-9 and -2 in the olfactory bulb following methyl bromide gas exposure [66].

### Epidemiological studies addressing methyl bromide exposure

**Figure 2:**
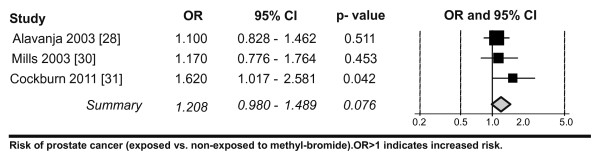
**Meta-analysis of cancer risk after exposure to methyl bromide**. The data showing all epidemiological studies clearly related to methyl bromide exposure (1990-2011) was analysed as described in the methods.

No epidemiological studies analysing the potential carcinogenic effects from the exposure to methyl bromide contaminants (or any other pesticide) due to its use in shipping and storage (i.e. in the atmosphere of containers) have been published to date. Most of the epidemiological studies analysing the causal link between methyl bromide exposure and the development of cancer have focused on the agricultural use of pesticides. However, the first clue implicating methyl bromide in a carcinogenic effect was from a study of chemical industry workers who were exposed to methyl halides. In this cohort study, an increased mortality from testicular cancer was reported in association with long-term occupational exposure to methyl bromide in a chemical plant [3]. There were only 3 more recent studies analysing the association between exposure to methyl bromide and cancer or toxicity. Two were cohort studies and one a case-control study. The main characteristics and results of the included studies are summarized in Table 2. All of them addressed exposure in relation to the use of methyl bromide in agriculture, either as occupational or environmental. One of the studies, the Agricultural Health Study (AHS), is a long-term cohort study of pesticide applicators and their spouses [28]. A report from the US National Cancer institute [35] stated that a few of the 45 evaluated pesticides showed evidence of a possible association with prostate cancer in the pesticide applicators. While methyl bromide was linked with the risk of prostate cancer in the entire group, exposure to six other pesticides was only associated with an increased risk of prostate cancer among those men with a family history of the disease [35]. Alavanja et al. reported a slightly increased relative risk among farmers occupationally exposed to methyl bromide [28]. This study demonstrated a gradient for the risk of prostate cancer with increasing level of exposure to methyl bromide, with the greatest risks among the two highest exposure categories (OR 3.47 95%-CI 1.37-8.76 for the highest exposure category) [28]. The risk was two to four times higher than for men who were never exposed to methyl bromide [28,35]. Among the 45 specific pesticides evaluated, only methyl bromide was associated with a statistically significant exposure-response trend. This effect was not seen among those without a family history of prostate cancer [35]. Mills and Yung also showed an association between methyl bromide exposure and prostate cancer with OR, 1.17; 95% CI (0.77-1.75), P = 0.45 although statistically non-significant [30]. Control subjects were age and location-matched farm workers without prostate cancer. The risk was associated with relatively high levels of exposure to methyl bromide. In a first study on prostate cancer and non-occupational exposure to pesticides, Cockurn et al. [31] confirmed the data from Alavanja et al. and provided evidence for an association between prostate cancer and the environmental exposure to methyl bromide in and around homes in highly agricultural areas [31]. Our meta-analysis shows a slight increase in prostate cancer risk after exposure to methyl bromide with OR, 1.21; 95% CI (0.98-1.49), P= 0.07. The results of the included studies are homogeneous (I^2 ^= 0%, thus we report results from the fixed effects model (see Figure 2, Table 2)). The model choice did not affect the results.

**Table 2:** Overview of epidemiological studies on methyl bromide effects (1990-2011)

Reference	Study design	Magnitude of study	Specified measure 1			Exposure to methyl bromide	cases			p value high vs. low
**study**	**year**			**sample size**	**location**		**cancer (prostate)**	**Odds Ratio adjusted**	**95% CI**	

[28]	2003	cohort study	occupational agriculture, farmers	55, 332	USA, IA, NC	exposed/controls *84/482	84	1.10	0.77, 1.36	0.004

						low exposure	6	2.73	1.18, 6.33	

						high exposure	5	3.47	1.37, 8.76	

[29]	2010	data analysis	occupational agriculture, farmers	55, 332	USA, IA, NC		5	3.47	1.37, 8.76	0.004

[30]	2003	case-control study	occupational agriculture, Hispanic farm workers	1, 332	USA, CA	exposed/controls 121/1110	64	1.17	0.77, 1.75	0.25

						low exposure	37	1.20	0.66, 2.18	

						high exposure	32	1.59	0.77, 3.30	

[31]	2011	case-control study	population, near intensive agricult. areas		USA, CA	exposed/controls 173/162	87	1.62	1.02, 2.59	0.1

						low exposure	45	1.81	1.03, 3.18	

						high exposure	42	1.45	0.82, 2.57	

[29]	2010	data analysis	occupational agriculture, farmers	55, 332	USA, IA, NC		5	3.47	1.37, 8.76	0.004

							toxic effects			

[32]	2006	cohort study	population, farmers' wives		USA, CA	exposed/controls *145/797		1.82	1.02-3.24	

A further epidemiological study [32] of intoxication cases showed an association with chronic low-dose methyl bromide pollution and chronic bronchitis with OR, 1.82; 95% CI (1,02-3.24), P = 0.04, due to non-occupational exposure.

### Related population-based epidemiological studies

Studies evaluating exposure to pesticides in general (i.e. without differentiating between compounds) have reported rather contradictory results, with some indicating an increase in cancer risk with risk increases ranging from 1.1 to 2.73 [9,67-70] and others showing rather lower cancer risks after pesticide exposure, ranging from 0.7 to 0.93 [67] for both workers and the community. Based on cohorts exposed to pesticides, 8 studies explored a possible association with increased cancer risk. Some reports identified an insignificant slightly decreased risk and others a significantly increased risk of cancer from pesticide exposure [9,68-70]. Yet, a declaration on carcinogenicity was not always available; similarly, retrospective personal or apocryphal reporting of product use (or misclassification of the degree of exposure or constitution of the chemical mixtures) is notoriously inadequate for risk association and assessments. Marusek et al. [39] concluded that this might also lead to misestimating of exposure level for control groups, especially when family members, generally considered as bystanders in farming activities, were used as controls. It has been reported that farmers tend to be at higher risk for cancers of the lip, brain, prostate, stomach, connective tissue melanoma and for carcinogenic changes in lymphatic and hematopoietic systems than the general population [71,72]. Several case-control studies have reported elevated relative risks of prostate cancer in agricultural workers [73]. Both in Italy and the USA [74-76], case control studies (though very inhomogeneous in nature) do report a slight increase in prostate cancer risk after pesticide exposure (with RR of 1.69 and RR 2.13). One US study reported a significantly increased risk of cancer in association with farming activities RR, 2.17; 95% CI (1.18-3.98), although the authors suggest a possible association with methyl bromide exposure, they acknowledged that another, as yet unidentified, factor may be involved [77]. More recent studies focused on cancer risk associated with pesticide use including methyl bromide: Issa et al. analysed two differently exposed groups of pesticide users in a retrospective study (1998-2006) [33]. To estimate prevalence differences between the two populations, directly exposed (farmers) and bystanders (farmers' wifes), the authors focused mainly on the change of habits, such as the use of the protective equipment or the applied dosage, concluding that there were some positive changes in the handling of pesticides amongst participants. The authors listed methyl bromide as one of the fumigant used but its possible carcinogenic effects were not addressed. A review by Weichenthal et al. [29] provided a comprehensive summary for most of the pesticides evaluated in the AHS.

The authors concluded that the data outside the study was still limited, but that the animal toxicity findings support the biological plausibility of a cancer risk. In addressing the issue of the link between the methyl bromide use and the incidence of the prostate cancer risk, the authors referred to the AHS study included in our meta-analysis and highlighted the increased risk of prostate cancer in methyl bromide applicators in the highest category of intensity-weighted exposure-days (Table 2).

### Bioavailability

The routes of absorption of methyl bromide are the lungs and skin with elimination routes via the lung, urine and faeces. The available animal biotransformation data in vivo show that seventy two hours after exposure to [^14^C]-methyl bromide, 43% was found in urine, ~40% was exhaled and 14-17% remained in the body (not only in fat tissue, but mainly in liver and kidneys). Notably, the animal data may not be directly extrapolated to humans (the serum half-life of bromide in humans is 12-16 days but only 1.5-3.5 days in the rat). Rats and mice metabolize methyl halides more rapidly than humans, so that the information on exposure concentration/duration and the association between the exposure concentration and symptoms cannot be directly extrapolated to humans. Fatal cases resulting from home fumigation exposure to humans were reported early [21]. One reported fatal case [13] provided both biomonitoring (exposure biomonitoring) and bioavailability data that showed initial serum methyl bromide levels on day 1 of 270 mg/L and of 29 mg/L on day 19 after exposure (at post mortem); the urine bromide concentration was 62 mg/L (normal <16 mg/L) one day after the exposure. Post-mortem (19 days after exposure) bromide levels were 17 mg/L in the bile, 24 μg/g in the liver and 28 μg/g in adipose tissue; urine formic acid was 58 μg/L (normal 50-360 μg/L). It needs to be noted that, as a consequence of the unrecognized first intoxication symptoms, the patient was presumed to have the flu and took bromide-containing flu medication. While this could have influenced the elimination kinetics, this data is important in highlighting differences between human and animal bioavailability.

### Exposure assessment and biomonitoring

On a short time scale, the assessment of possible methyl bromide intoxication can be performed by air (ambient) monitoring or exposure biomonitoring [78]. Ambient monitoring data, associated with intoxication incidents, revealed values of 2-10 ppm methyl bromide in storage units (measured in cold-storage facilities, where off-gassing grapes were stored) [10]. We have measured over 4000 import freight container units in Hamburg and Rotterdam (2007-2010) and found the following range of methyl bromide concentrations in air samples from containers arriving at the harbor customs for inspection: 0.005-50 ppm (11.5% incidence in 2006-2008) and 0.005-7.1 ppm (4.8% in 2009/2010) [18,19]. In 2006, 3 individual container atmospheres had methyl bromide levels exceeding 800 ppm [17]. It has to be noted that the container air samples had multiple contaminations with fumigants and/or toxic industrial chemicals (like benzene) [17-19,79].

If supported by toxicological validation, exposure assessment based on biomarkers [78,80] provides the most valuable information about possible methyl bromide intoxication (for the individual incorporation through the lungs and skin). with the parent methyl bromide, or its metabolite bromide, being used for the biomonitoring of methyl bromide exposure. In a 17-year follow-up study, urinary bromide concentrations in factory workers (using protective equipment) exposed to methyl bromide were 25.2 ± 18.7 mg/g creatinine (3.0-125 mg/g creatinine) [20]. The measured urine values of 32.4-68.7 mg bromide/mg creatinine and serum levels of 36.2-52.1 mg bromide/L (normal reference levels are <5 mg/L) were associated with technical incidents and could be correlated with reported episodes of dizziness [20]. Blood samples from greenhouse workers analyzed 11 days after the application of methyl bromide revealed 3.4-20.6 mg/L of serum bromide. The increased bromide values, observed in most applicators, were associated with reported symptoms of irritation to the eyes, coughing, neurological, psychiatric, respiratory and gastro-intestinal symptoms [11]. Biological effect biomonitoring [78,80] provides useful information about prior intoxication and has implied an association between an increase in proximate pre-carcinogenic lesions after pesticide exposure and the cancer risk [81-83]. A prospective analysis of blood samples from more than 6700 agricultural and greenhouse workers revealed an elevation of cytogenetic biomarkers and enhanced cancer risk after pesticide exposure [81]; Several other studies using micronuclei (and other functional cytogenetic biological markers) revealed both an increase in cytogenetic damage after exposure to pesticide mixtures and their correlation with an increased cancer risk in several European populations [74,82,83].

### Reference values, community exposure limits

The calculated reference concentration values (RfC) for non-carcinogenic effects of methyl bromide in humans [84] can be regarded as community exposure limits. The RfC is a reference point to gauge potential effects, the incidence of which increases for an exposure greater than RfC [45]. An RfC limit value of 0.210 ppm (0.210 mL/m^3^) was recently estimated for acute inhaled exposure of methyl bromide [84]. Also, for a subchronic exposure to methyl bromide for 1 week, the RfC was estimated to be 0.129 ppm and 0.079 ppm for adults and children, respectively; while the chronic 6 week RfCs were estimated to be 0.002 ppm and 0.001 ppm for adults and children, respectively. The California Office of Environmental Health has also settled non-cancer reference dose (RfD) values for acute air exposure to methyl bromide at 0.05 ppm (neurologic targeted toxicity) and for chronic RfD for the respiratory tract target (based on degenerative and proliferative lesions of the olfactory epithelium of the nasal cavity) to be 0.005 mg/m^3 ^(0.0012 ppm) [9]. Additionally, community exposure data, which showed air values of 0.005 ppm [9,69,85] due to pollution from farming activities, provides the basis for the estimation of hazard quotients (HQ) (defining non-cancer risk) [84,85]. These risk quotients were characterized for populations within a few miles of the air monitoring stations [9]. The HQ is defined as a ratio between the estimated intake of methyl bromide (in mg/kg/day) and the reference dose (RfD); the acute HQ was estimated to be 0.7 mg/kg/day (95% CI), the subchronic as 13.9 mg/kg/day (95% CI) and the HQ for chronic intake as 2.0 mg/kg/day [84].

## Discussion

The halogenated hydrocarbon pesticide methyl bromide, which was designed for phase-out in 2005, remains in frequent use because of various critical use exemptions and new regulations. The exposure assessment data and epidemiological analysis indicate health risk concerns for both workers and the general public [31,32]. Recent case reports continue to demonstrate episodes of illness (with disabling neurological symptoms, memory difficulties and dizziness) in association with elevated levels of serum bromide [10,15].

Methyl bromide is at least as poisonous to humans as it is to the pests with genetic susceptibility (i.e. the conjugator status) or acquired single point mutations playing an essential role in humans. The conjugator status varies phenotypically between species and individuals and may help to explain the variation in toxicity observed (with data showing no immediate, otherwise expected, effects). In human non-conjugators, the absence of the glutathione S-transferase (GST) pathway pushes methyl bromide into alternative oxidation pathways [43], effectively reducing its acute neurotoxicity but concomitantly and insidiously exacerbating its chronic genotoxic effects [40-42].

The exposure to pesticides in agriculture is almost always additive in nature [35]. The possible additive or subadditive effects might be different for cases of exposure to fumigated container and contaminated goods however. We found not only methyl bromide but also high levels of contamination with ethylene dichloride, methylene chloride, ethylene dibromide or tetrachlorethanes in import containers (all halo-methanes or halo-ethanes that share signalling pathway disruption mechanisms). Many epidemiological studies refer to pesticide exposure but without discriminating between the different chemical entities nor their formulations, which differ not only chemically but also in their toxicity, patho-physiological mode of action, target organ, symptoms and possible carcinogenic status (with many not listed as carcinogenic nor even evaluated [35]. Retrospective personal or apocryphal reporting of product use, or misclassification of the degree of exposure or constitution of the chemical mixtures, all fail to contribute adequately to risk associations and assessments. Occupational circumstances associated with farming alone (as confounder) do not appear to provide a risk factor for prostate cancer; rather there is a perceived decrease in overall cancer incidence among unexposed farmers [86]. On the other hand, the community exposure risks to airborne agricultural pesticides have been documented [9] and the study from Cockburn et al. demonstrated an association between prostate cancer and the ambient non-occupational exposure to methyl bromide [31].

Our meta-analysis indicates an increased prostate cancer risk after exposure to methyl bromide. The International Agency for Research on Cancer (IARC) continues to classify methyl bromide in the carcinogenic category 3 (defined as unclassifiable as to its carcinogenicity to humans because of inadequate evidence in humans and limited evidence in experimental animals) [87]. Yet many studies provide evidence that application of this pesticide may not only elicit a number of toxic effects but also is associated with an increased risk of cancer [28-31]. However, the carcinogenicity of methyl bromide cannot be easily explained as a function of the concentration levels and the exposure period, especially with the limitations and disputed relevance of animal experimentation. More recent data delineate the role of single point mutations in enhanced prostate cancer risk after pesticide exposure, affecting genes which code for phase I/II and oxidative stress enzymes, [55].

The complicated and complex biotransformation pathways of methyl bromide in humans have only been partially elucidated. Human studies are rare and any extrapolation from animal data is difficult to justify. Further investigations are needed to explore the molecular mechanisms of the toxicological and carcinogenic effects of methyl bromide in more detail.

The exposure misclassification in many epidemiological studies may have caused an underestimation of the effects (especially when the control groups, such as family members, are also exposed). It has also to be emphasized that many available studies concern average risks and, therefore, do not represent the actual risks in genetically predisposed human subjects. We recommend further studies to redress this deficiency.

## Conclusions

Both the epidemiological evidence and toxicological data suggest a link between methyl bromide exposure and serious health problems, including cancer risk (prostate cancer), from occupational and community exposure. The carcinogenic classification of methyl bromide should be reevaluated.

## List of abbreviations

AHS: Agriculture Health Study; CUE: critical use exemtions: DNA: Deoxyribonucleic acid: EPA: Environmental Protective Agency; GSH: gluthatione ((2S)-2-amino-4-{[(1R)-1-[(carboxymethyl)carbamoyl]-2-sulfanylethyl]carbamoyl}butanoic acid); GST: Gluthatione S-transferase;GSTT1: Glutathione S-transferase theta;HQ: hazard quotient; IUPAC: International Union of Pure and Applied Chemistry; NTP: National toxicology program OD: odds ratio; ppm: parts per million (= mL/m^3^); RfC: Reference concentration values; SNP: single nucleotide polymorphism.

## Competing interests

The authors declare that they have no competing interests

## Authors' contributions

XB and LTB made substantial contributions in the conception, design of the study and the interpretation of data. SK did the detailed literature search and building up the molecular model. LTB analyzed the toxicological data and MVG performed the analysis of epidemiological data and performed the meta-analysis. LTB wrote the manuscript. All authors approved the final version for submission.
